# Does healthcare consumerism serve as a barrier or facilitator to the implementation of value-based primary care? Strategies to promote synergy and success

**DOI:** 10.3389/fmed.2023.1269796

**Published:** 2023-09-01

**Authors:** Pavani Rangachari

**Affiliations:** Department of Population Health and Leadership, School of Health Sciences, University of New Haven, West Haven, CT, United States

**Keywords:** value-based care, consumerism, primary care, retail healthcare, patient-provider trust, consumer engagement, healthcare technology, health equity

## Abstract

**Introduction:**

Value in health care is described as the measured improvement in a patient’s health outcomes for the cost of achieving that improvement. In the United States, value-based care has been heralded by providers, payers, and policymakers alike, as a path to addressing the challenges facing the healthcare system and achieving the aspirational goals of the Quadruple Aim of healthcare. Primary care is often viewed as the foundational cornerstone for implementing value-based care. However, primary care is also considered as ground-zero for the rise in healthcare consumerism.

**Methods:**

In essence, consumerism refers to increasing expectations from patients (consumers) to be more active participants in decisions related to their healthcare. While much of the literature has portrayed the rise in consumerism as a barrier to the implementation of value-based primary care, some have argued that it may have potential to synergize with and facilitate the implementation of value-based primary care. This paper applies an enhanced stepwise implementation framework for value-based (equitable) care, to examine the potential for conflict and synergy between consumerism and value-based care in the emerging retail model of primary care. The application is based on the potential actions of four key stakeholder groups: (1) retail healthcare entities, (2) primary-care providers, (3) consumers (patients), and (4) healthcare payers.

**Results:**

The analysis helps to articulate the responsibilities of each stakeholder group in ensuring synergy between consumerism and value-based primary care. In addition, it helps to identify three drivers of synergy between consumerism and value-based care: (1) trust in the patient-provider relationship, (2) connected consumer-centric technology solutions, and (3) value-based consumer-centric payment models.

**Discussion:**

Overall, the application helps to articulate a comprehensive framework for implementing value-based care that incorporates both the principles of consumerism and active consideration for health equity.

## Introduction

Leading frameworks conceptualizing healthcare systems agree that effective healthcare systems must produce better patient experience and health outcomes at a sustainable cost (1–3). Additionally, health equity, or the fair distribution of health outcomes within populations, has been embraced by the World Health Organization (WHO) as a primary aim (4, 5). Although countries around the world struggle to achieve these aims, the United States (US) is known to have the most costly and sub-specialized healthcare system, with poorer health outcomes and health equity at a population level, compared to any other industrialized peer nation (6, 7).

In recent years, value-based care has been put forth as an approach for addressing the challenges facing the US healthcare system (3, 8, 9). Value in health care has been described as the measured improvement in a patient’s health outcomes for the cost of achieving that improvement (9). Since value is created only when health outcomes improve, it has been argued that value-based care cannot be defined purely in terms of cost reduction (8, 9). Notably, value-based care has been heralded by providers, payers, and policymakers alike, as a path to achieving the aspirational goals of the Quadruple Aim of healthcare, which entails improving the patient experience, improving the health of populations, and reducing the per capita cost of health care, while also improving the clinician’s experience of providing care (10).

Nonetheless, formidable barriers exist to the widespread, successful implementation of value-based care, including the slow pace of change of payer reimbursement models and provider resistance to redesigning care delivery models (11–13). In recent years, the emerging trend of consumerism in healthcare has received considerable attention for its potential to serve as a barrier to value-based care, although some have argued that consumerism has the potential to synergize with and facilitate the implementation of value-based care (13, 14). In essence, consumerism refers to increasing expectations from patients (consumers) to be more active participants in decisions related to their healthcare (15, 16). Based on this interpretation, there may be no reason to view consumerism as a barrier to value-based care. If anything, it could be viewed as a facilitator, if both parties (patients and providers) have a shared goal of improving health outcomes (14). However, if consumerism is interpreted as the “commodification” of healthcare, whereby consumers expect healthcare to function like any other service (e.g., restaurants), with a focus on outcomes that may arguably be different from what providers value (e.g., convenience or speed), then consumerism could be viewed as a barrier to the implementation of value-based care (13).

### Purpose of this paper

Primary care is viewed as a foundational cornerstone for value-based care since the four primary care core functions (4Cs) of contact (access), continuity, comprehensiveness, and coordination are each associated with improved health outcomes (11). However, primary care is also viewed as ground-zero for the rise in consumerism, e.g., the “retail primary care consumer” who is willing to “shop” for primary care services within large integrated healthcare marketplaces (13).

This paper explores the potential for conflict and synergy between consumerism and value-based care in the emerging retail healthcare model of primary care. It begins by articulating an enhanced framework for implementing value-based care that also incorporates active considerations for health equity. The framework is applied to the retail model of primary care to discuss potential avenues for conflict and synergy between consumerism and value-based care, based on the actions of four stakeholder groups: retail healthcare entities, primary care providers, consumers, and payers. The analysis helps to identify strategies for mitigating conflict and promoting synergy between consumerism and value-based care, to ensure the success of value-based care models in primary care.

### An enhanced framework for implementing value-based care that incorporates active consideration for health equity

By focusing on the outcomes that matter most to patients, value-based care aligns care with how patients experience their health. Population health only improves when the health outcomes of many individuals with shared health needs improve, which is the focus of value-based health care. Likewise, by organizing teams to care for individuals with similar needs, a value-based approach enables expertise and efficiency, to drive costs down. Measured health outcomes in turn demonstrate clinicians’ ability to achieve results with patients and families and drive improvement in the results that matter most to both patients and clinicians. Correspondingly, value-based health care puts decisions about how to deliver care in the hands of the clinical team, supports their professionalism, and the power of clinician-patient relationships, to deliver effective and appropriate care.

Following years of research on value-based care, medical academic literature has articulated a five-step framework that healthcare organizations could use to implement value-based care: (1) understanding shared health needs of patients; (2) designing a comprehensive solution to improving health outcomes; (3) integrating learning teams; (4) measuring health outcomes and costs; and (5) expanding partnerships (9). It is noteworthy however, that this framework does not incorporate explicit considerations for health equity.

The COVID-19 pandemic served to both expose and exacerbate health disparities in the United States (17). Value-based care and payment models are known to have the potential to reduce health disparities (17, 18). During the pandemic, organizations that received a greater proportion of prospective (value-based) payments were protected, since their revenues were less affected by reductions in service volume. Moreover, value-based payment models encouraged organizations to develop partnerships and invest in infrastructure to address people’s clinical and social needs. Correspondingly, these organizations had greater success in adapting to the public health emergency with new care models to maintain continuity of care when faced with a substantial shift to telehealth and reduction in elective services (17, 18).

At a national level however, few organizations are explicitly prioritizing health equity in their value-based care or payment models. To address this concern, health policy advocates have put forth several strategies for providers and payers to ensure consideration for equity in value-based care, including (1) the selection of equity-focused quality measures, (2) adjusting performance measures for social risk to address health disparities, and (3) empowering healthcare organizations to address social drivers of health (18). Based on these policy recommendations, it would be reasonable to argue that each of the five steps of the framework for implementing value-based care could be enhanced to incorporate active considerations for health equity, e.g., Step 1 could be enhanced to “understanding shared health needs of patients, with active considerations for health equity,” and so on and so forth.

### Applying the framework to examine the potential for conflict and synergy between consumerism and value-based primary care

Applying the enhanced framework on value-based (equitable) care to primary care must begin with a recognition of the primary care context, including: (1) the current crisis in traditional primary care in the United States, (2) the rise of consumerism and growing threat of new retail healthcare market entrants, and (3) the urgent call for primary care providers to reclaim their territory through concerted efforts to implement value-based care.

Despite the rising momentum toward paying for value, healthcare financing continues to focus heavily on payment for transactional, visit-based care (e.g., the Medicare Relative Value Unit schedule). This leaves few options for primary care practices to provide high-value services (e.g., between-visit preventive care, care coordination, and chronic disease management) due to lack of reimbursement. However, with healthcare expenditures continuing to outpace economic growth, pressures have mounted on primary care to provide these services. The growing expectations for high-value services coupled with low revenue streams in turn, has resulted in many primary care practices struggling to maintain financial sustainability (11).

At the same time, there is evidence of rising consumerism in primary care, a trend that has resulted in growing threats to traditional primary care by new market entrants seeking to promote retail healthcare tactics in the healthcare space. Examples include nonhealthcare businesses entering healthcare (Amazon, Google, etc.), national pharmacy chains, medical device and pharmaceutical firms, information technology companies, startups, as well as existing organizations, insurance companies, integrated delivery systems (e.g., Kaiser Permanente). These market changes reflect the reality that so long as traditional primary care fails to adequately meet patients’ expectations and needs, new entrants will attempt to fill this void (11, 13).

In recognition of the escalating crisis in primary care, medical leaders have called upon primary care providers to adopt new models of care delivery that reinforce the potential for improving value. These leaders have argued that redesigning care delivery and payment models based on the following principles will lead to higher value, which in turn will necessitate new approaches to workforce training: (1) reward for value, including between-visit preventive care. (2) focus on building relationships with patients (consumers) through teams and technology, with non-physicians assuming an increasing role in healthcare. (3) focus on high complexity presentations by generalist physicians, and (4) focus on whole-person care that addresses health behaviors and provides vision, hearing, dental, and social services (11).

The urgent need for concerted efforts from primary care providers to implement value-based care in turn helps to underscore the need for effective strategies to mitigate conflict and promote synergy between consumerism and value-based primary care. Notably, the actions of four stakeholder groups involved, (1) retail healthcare entities, (2) primary care providers, (3) consumers, and (4) payers, have potential to affect the interplay between consumerism and value-based care. In the sections below, the framework for implementing value-based (equitable) care is applied to examine the potential for conflict and synergy between consumerism and value-based primary care, arising from actions of each stakeholder group. The application helps to identify strategies for promoting synergy between consumerism and value-based primary care.

### Retail healthcare entities

Retail healthcare entities could engage in a variety of activities that foster conflict between consumerism and value-based care (13, 14).

1. They could allow consumers (patients) to comparison-shop by delivering greater price transparency, which in turn has the effect of creating price competition, pressuring sellers to lower the prices for their services.
2. They could also engage in “volume selling” or getting consumers to access lower-cost, health services (e.g., wellness services, fitness monitoring, walk-in clinics) on a frequent basis, thereby emphasizing the purely transactional aspects of healthcare delivery.
3. They may also engage in market segmentation, i.e., grouping patients with similar needs and preferences to target certain services, a tactic that allows the retail entity to undercut the provider and approach the patient directly to market products or services, e.g., wearable devices, that the patient may not need, but may in fact place greater demand on the provider’s time with uncertain reimbursement.

In summary, a consistent theme in retail healthcare tactics is the introduction of intermediaries between the buyer (consumer/patient) and seller (provider) to shape decisions about which products consumers should buy, thereby undermining providers’ ability to build meaningful relationships with the patient (13, 14) (Table 1).

**Table 1:** Potential for conflict and synergy between consumerism and value-based care arising from actions of retail primary care companies.

Framework for implementing value-based (equitable) care	Potential for conflict through stakeholder actions	Potential for synergy through stakeholder actions
(1) Understanding shared health needs of patients, with active consideration for health equity.	Comparison-shopping (price competition), volume-selling, and market segmentation are all retail tactics that can be detrimental to the provision of value-based care by pitting the consumer against the provider and by introducing the retail entity as an intermediary in the provider-patient relationship (13), which in turn can prevent providers from being able to develop an understanding of shared health needs of patients.	Some retail healthcare entities have chosen to serve as low-cost extensions to primary care as opposed to substitutes. They are staffed by nurse practitioners to manage acute conditions and return patients to their primary care physician (14). This approach enables primary care providers to take the lead in understanding shared health needs of patients and relying on retail entities to maintain continuity of care through efficient use of resources.
(2) Designing a comprehensive solution to improving health outcomes, with active consideration for health equity.	Volume-selling shifts the focus to transactional excellence, whereas designing a comprehensive solution to address health needs requires relational excellence between providers and patients (13) to craft a solution that fits within the patient’s life context, addresses medical and social needs, and is efficient in the use of resources.	By partnering with providers (instead of competing with them), retail healthcare entities can help to maintain continuity of care and be part of a comprehensive solution for addressing patients’ medical and social needs.
(3) Integrating learning teams, with active consideration for health equity.	By competing, instead of collaborating with providers to address patients’ needs, retail entities hinder providers’ ability to establish co-located learning teams to coordinate care and improve outcomes with efficient use of resources.	Some retail care entities are also committed to sharing data about patient care encounters with primary care physicians, and some are committed to helping patients without primary care find a medical home (14). Such approaches enable service integration to ensure effective and efficient care. They also enable retail entities to work as a team with primary care providers, to integrate learning and improve health outcomes.
(4) Measuring health outcomes and costs, with active consideration for health equity.	Market segmentation undermines providers’ ability to work in the best interests of the patient by allowing the retail entity to target products and services to patients that the provider may deem as inappropriate or deviating from evidence-based guidelines for treatment. This in turn may hinder the development of health outcome and equity measures that are valued by providers.	Studies indicate that care provided at retail clinics for common acute illnesses, e.g., respiratory infection, urinary tract infection, is as good if not better than care delivered in ambulatory or emergency room settings (22, 23). Also, many of these entities are committed to strict evidence-based guideline adherence, which in turn is directly aligned with the value-based care goals of measuring and improving outcomes (14, 22).
(5) Expanding partnerships, with active consideration for health equity.	Pitting consumers against providers through volume selling and market segmentation hinders the development of a collaborative spirit among providers, which in turn is essential for expanding partnerships within the profession and the community, to improve health outcomes of people with similar needs and promote population health.	Retail strategies of serving as a low-cost extension of primary care, using a team-based approach to data sharing, and implementing evidence-based guidelines to improve outcomes, can enable primary care providers to expand partnerships to address needs of groups of patients, to promote population health and achieve the goals of value-based care.

### Primary care providers

Primary care providers who do not embrace value-based care and continue to espouse fee-for-service may view retail clinics as a source of competition. These providers may see retail clinics as skimming easier cases and leaving the more complex and time-consuming patients for the primary care providers, thereby adding new challenges to their workflow in the broader context of lack of reimbursement for care coordination and other high-value services (13, 14). In this scenario, some primary care providers may respond by establishing “direct primary care” models, i.e., stand-alone practices that no longer deal with insurance and instead require a smaller panel of patients to pay monthly subscription fees to receive more on-demand care (14) (Table 2).

**Table 2:** Potential for conflict and synergy between consumerism and value-based care arising from actions of primary care providers.

Framework for implementing value-based (equitable) care	Potential for conflict through stakeholder actions	Potential for synergy through stakeholder actions
(1) Understanding shared health needs of patients, with active consideration for health equity.	Direct primary care models are exclusive practices that have potential to limit access to primary care for those without the means to contract directly. This in turn greatly limits the ability to understand shared health needs of patients within a population (14). Correspondingly, this model has potential to exacerbate disparities, as the most vulnerable would face the greatest challenges for access.	Primary care providers who embrace value-based care principles are likely to view retail sites as valuable low-cost extensions of primary care that could be leveraged as partners for improving outcomes at a sustainable cost. Such providers are also likely to invest efforts in building trusting relationships with their patients (consumers) to understand the full spectrum of their medical and social needs for developing holistic and effective care plans.
(2) Designing a comprehensive solution to improving health outcomes, with active consideration for health equity.	By making their practices exclusive and competing with retail care models and other primary care providers, direct primary care providers can greatly limit the ability to design a comprehensive solution for improving health outcomes and promote population health.	Embracing value-based care, establishing partnerships with retail care sites, and investing in consumer-centric technology like telehealth and remote (home-based) diagnostics and monitoring, can engage and empower consumers to be stewards of their own health, while also catering to the growing consumer demand for convenience and efficiency (15, 16). The increasing potential for connectivity between remote technology and physicians’ offices, moreover, helps to design a comprehensive solution for improving health outcomes. Moreover, remote care can reduce the need for office visits and alleviate transportation barriers and costs, thereby helping to promote equity in healthcare access and outcomes.
(3) Integrating learning teams, with active consideration for health equity.	Direct primary care models can have the effect of propagating silos by creating exclusive practices and mitigating the potential for learning, collaboration, and care coordination needed to improve health outcomes at a sustainable cost.	Partnering with retail care sites that serve as low-cost extensions of primary care and sharing data on patient care encounters can help to integrate learning teams and improve care coordination. Similarly, increasing connectivity between remote (home-based) care technology and physicians’ offices helps to integrate learning teams and remain continuously responsive to patients’ evolving needs.
(4) Measuring health outcomes and costs, with active consideration for health equity.	Direct contracting creates silos (exclusive patient panels) and limits the potential for data sharing with other primary care providers, which hinders the ability to measure health outcomes and costs and reduce health disparities.	Primary care providers that espouse value-based care principles, can initiate meaningful partnerships with retail care sites, patients/families, and community partners (11, 14), to implement evidence-based guidelines, and ensure data sharing and connectivity, to measure and improve health outcomes and promote health equity.
(5) Expanding partnerships, with active consideration for health equity.	Direct primary care models could adversely impact primary care providers who do not engage in direct contracting, by forcing them to care for a sicker and more vulnerable population (14), thereby limiting their ability to improve outcomes, manage population health, and expand partnerships.	Primary care providers who strive to redesign care to maintain continuity, establish retail care partnerships, invest in telehealth services, and leverage the potential of connected remote care technology, will be better poised to expand partnerships for improving health outcomes for the populations they serve, at a sustainable cost.

### Healthcare consumers

Ultimately, health outcomes need to improve for value-based care to succeed, and consumers can derail success through no-shows or non-adherence resulting from lack of trust and engagement. The concept of “Patient” implies responsibility on the part of providers, and a historical hierarchy based on the premise that providers act or decide on behalf of patients. The concept of “Consumers,” on the other hand, is founded on the principle of choice and preferences (19). Consumers make decisions that affect their health based on information they choose to pay attention to. Therefore, blind faith in providers’ actions may no longer be the norm, which in turn forms the root of trust issues that are increasingly being recognized as a disruptive force in the US healthcare system (20). Value-based care is critically dependent on “patient engagement.” Patients must desire to improve their conditions while using resources responsibly (9). Therefore, providers need to factor in holistic information related to people’s lives, needs, preferences, technology use, and constraints into care plans to build trust and ensure patient engagement (Table 3).

**Table 3:** Potential for conflict and synergy between consumerism and value-based care arising from actions of consumers.

Framework for implementing value-based (equitable) care	Potential for conflict through stakeholder actions	Potential for synergy through stakeholder actions
(1) Understanding shared health needs of patients, with active consideration for health equity.	When consumers do not trust their healthcare providers and decide to use the system infrequently or only for sick care, it could become challenging for providers to understand patients’ medical and social needs (19, 20).	Value-based care is critically dependent on patient trust and engagement. Correspondingly, providers need to invest considerable effort in understanding patients’ shared medical and social needs to develop holistic care plans to engage and empower patients to improve outcomes at a sustainable cost.
(2) Designing a comprehensive solution to improving health outcomes, with active consideration for health equity.	Consumer health illiteracy, and lack of trust in the healthcare system could prompt consumers to seek information for self-care from social media or private data platforms elsewhere (19) and only access the healthcare systems minimally, which could greatly hinder the ability to design a comprehensive solution	Adoption of consumer health technology is growing fast. Wellness is a growing priority in people’s lives and the use of mobile health apps and consumer wearables is advancing rapidly (19). Developing effective channels of communication with patients to gain a broad understanding of consumers’ use of health information and technology can help to design a comprehensive solution for improving outcomes in partnership with patients.
(3) Integrating learning teams, with active consideration for health equity.	If providers remain unaware of consumers’ use of technology for self-care, they may find it challenging to remain connected with their patients and to integrate learning teams for improving outcomes.	Earning patients’ trust will enable better information and data sharing from patients to facilitate a clearer understanding of patients’ needs which in turn will help to integrate learning teams. Building trust with patients and families in turn will require providers to convince people that they are placing the patients’ best interests above any self-interest of their own.
(4) Measuring health outcomes and costs, with active consideration for health equity.	Poor provider-patient communication resulting from mistrust and misinformation hinders the ability to capture meaningful data about patient needs and health behaviors which in turn limits the capacity to measure health outcomes and costs.	Providers must factor in information about people’s medical needs and living conditions (social health) into care plans and strategies. This type of holistic approach to patient care will help to build trust and facilitate information-sharing regarding consumers’ use of healthcare technology that providers in turn could leverage to measure and improve outcomes at a sustainable cost.
(5) Expanding partnerships, with active consideration for health equity.	Ineffective patient-provider communication can create misinformation related to a patient’s health needs making it a challenge to achieve the goals of value-based care of improving health outcomes at a sustainable cost. Since expansion of partnerships is contingent on improving outcomes, this step is hindered by the absence of trusting relationships between providers and patients.	An engaged and empowered consumer base can serve as a strong foundation for effective information sharing about patients’ health and social needs to integrate learning teams and measure and improve outcomes which in turn will enable the expansion of partnerships to widen the base and influence of value-based care.

### Healthcare payers

Public and private healthcare payers have a key role to play in regulating the interplay between consumerism and value-based care. Until recently, value-based payment models focused exclusively on influencing providers to reduce costs, which in turn had the effect of creating perverse incentives among providers to cherry pick healthier patients to demonstrate outcomes improvement at reduced costs (21). The increasing priority on health equity, however, has potential to facilitate a more holistic approach to value-based care by encouraging providers and consumers to work together to improve outcomes and reduce disparities, with efficient use of resources (17, 22). When payers’ efforts focus exclusively on inducing providers to make cost-effective clinical decisions or on influencing consumers’ healthcare purchasing behavior, the goals of value-based care could become difficult to achieve. For value-based care to succeed, providers and consumers need to share the responsibility for effective health care, i.e., improving health outcomes, and promoting health equity at a sustainable cost (Table 4).

**Table 4:** Potential for conflict and synergy between consumerism and value-based care arising from actions of payers.

Framework for implementing value-based (equitable) care	Potential for conflict through stakeholder actions	Potential for synergy through stakeholder actions
(1) Understanding shared health needs of patients, with active consideration for health equity.	Payment models designed exclusively to influence providers to reduce costs can create perverse incentives to cherry-pick (21) healthier patients which can serve as a barrier to understanding the shared health needs of patients to improve outcomes and promote population health.	Designing value-based payment models with a focus on improving outcomes and health equity at a sustainable cost, has the potential to motivate providers to understand shared health needs of groups of patients with a view to improving outcomes and health equity at a sustainable cost.
(2) Designing a comprehensive solution to improving health outcomes, with active consideration for health equity.	Payment models that are focused on influencing consumers’ healthcare purchasing behavior (e.g., incentives to purchase wellness services or wearables) without provider buy-in, can adversely impact the provider-patient relationship (19) and hinder provider engagement in seeking comprehensive solutions to improve health outcomes.	Value-based payment models that support consumer-centric care options like low-cost, convenient, retail care (14), and connected, remote (home-based) care technology (16), can empower providers to address both medical and social drivers of health to design a comprehensive solution for addressing shared health needs of patients to improve health outcomes and health equity.
(3) Integrating learning teams, with active consideration for health equity.	Payment models that are designed to reward cost reduction as opposed to the intended goals of value-based care (16), have the potential to induce providers to focus on cost cutting (at the expense of outcomes improvement), which in turn could impede with the integration of learning teams for improving outcomes.	Payment models that focus on improving outcomes and reducing health disparities, have the potential to unite providers and patients in seeking a comprehensive solution to address health needs through data sharing and remote technology-connectivity, to integrate learning teams for success.
(4) Measuring health outcomes and costs, with active consideration for health equity.	Payment models that focus on cost reduction may result in little or no attention to measuring and improving outcomes, thereby hindering the ability to achieve the goal of value-based care.	Payment models that stay true to the philosophy value-based care of improving outcomes can enable data sharing across collaborating entities, effective integration of learning teams and measurement of outcomes, costs, and health equity to ensure attainment of the goals of value-based care.
(5) Expanding partnerships, with active consideration for health equity.	Payment models focused on cost reduction or on influencing consumers’ purchasing behavior, may pit providers against patients, and prevent the development of a provider-patient partnership for improving outcomes, which in turn could hinder the ability to expand partnerships without a sufficient evidence base on improved outcomes to appeal to diverse pool of stakeholders.	Payment models that incentivize demonstration of improved health outcomes at a sustainable cost, could enable providers to be successful in expanding partnerships to improve outcomes for groups of people with similar needs, and promote population health and equity, to achieve the goals of value-based care.

## Discussion

The Health Care Transformation Task Force has articulated the primary goal of consumerism as: “*supporting a person’s ability to receive high-quality healthcare that best aligns with their goals, expectations, and preferences for services in a culturally relevant way. Reduction in cost, while important, should be considered a secondary benefit* (15).” The analysis in this paper helps to identify the responsibilities of four key stakeholder groups in enabling the principles of consumerism to be synergistically incorporated into value-based care models.

Retail healthcare entities must partner with primary care providers to serve as low-cost, convenient, and effective extensions of primary care, and ensure a high degree of data sharing and connectivity with primary care practices to promote synergy between consumerism and value-based care. Primary care providers in turn, must embrace the principles of value-based care, and partner with retail care sites to offer consumer-centric options for acute care. Concurrently, they must strive to build strong, trusting relationships with patients, to create a foundation for designing comprehensive solutions to address patients’ health needs, to improve health outcomes and equity at a sustainable cost. Consumers can derail the success of value-based care by not showing up for appointments, not sharing information, using disconnected healthcare technology, and not adhering to care plans, owing to lack of trust and engagement. Correspondingly, payers must design payment models that reward consumer-centric value-based (equitable) care options to promote trusting relationships between providers and patients and facilitate a shared responsibility for improving health outcome and equity at a sustainable cost, to ensure the success of value-based primary care.

The analysis and discussion in this paper also help to identify three key themes among the drivers of synergy between consumerism and value-based care:

1. Trust in the provider-patient relationship. To build trust, primary care providers must convince patients that they are placing their best interests above their own self-interest; and demonstrate respect for differing patient beliefs and perspectives by engaging in dialogue to provide alternate information and recommendations (20).
2. Connected consumer-centric healthcare technology solutions. Consumer-centric remote (home-based) diagnostics and monitoring technology (14, 16) that are connected to primary care practices can serve the dual purpose of incorporating both principles of consumerism (e.g., convenience and efficiency) and consideration for health equity (e.g., elimination of transportation barriers) to achieve the goals of value-based primary care.
3. Value-based consumer-centric payment models. These type of payment models may be indispensable in promoting trusting relationships between patients and providers and facilitating a shared responsibility for designing comprehensive solutions to address health needs of groups of patients, and improving health outcomes and equity, at a sustainable cost, to achieve the goals of value-based care (16, 19).

Overall, the analysis helps to articulate an enhanced, comprehensive framework for implementing value-based care that incorporates both the principles of consumerism and consideration for health equity. In other words, each step of the five-step framework for implementing value-based care (discussed earlier) (9), can be enhanced to incorporate both principles of consumerism and consideration for health equity. For example, Step 1 could be modified to “understanding shared health needs of patients, with active consideration for the principles of consumerism and health equity.”

## Conclusion

This paper applies a stepwise framework for implementing value-based (equitable) care to examine the potential for both conflict and synergy between consumerism and value-based care in the retail model of primary care. The analysis helps to articulate the responsibilities of four stakeholder groups and underscore the importance of (1) trust in the patient-provider relationship, (2) connected consumer-centric technology solutions, and (3) value-based consumer-centric payment models in promoting synergy between consumerism and value-based primary care.

As the healthcare industry continues to shift to value-based care, and consumerism rises, strong, trusting provider-patient relationships that foster a shared responsibility for designing comprehensive solutions to address health needs, will hold the key to success. Concurrently, a convergence between payers and providers will be required to deliver on the expectations of value-based, consumer-centric payment models. In this scenario, investments in consumer-centric technology that facilitates connectivity across the four stakeholder groups (retail healthcare entities, providers, consumers, and payers), has potential to serve as a foundational cornerstone for attaining the goals of consumer-centric value-based primary care with active considerations for health equity.

## Data availability statement

The original contributions presented in the study are included in the article/supplementary material, further inquiries can be directed to the corresponding author.

## Author contributions

PR: Conceptualization, Investigation, Validation, Writing – original draft, Writing – review and editing.

## Funding

The author(s) declare that no financial support was received for the research, authorship, and/or publication of this article.

## Conflict of interest

The author declares that the research was conducted in the absence of any commercial or financial relationships that could be construed as a potential conflict of interest.

## Publisher’s note

All claims expressed in this article are solely those of the authors and do not necessarily represent those of their affiliated organizations, or those of the publisher, the editors and the reviewers. Any product that may be evaluated in this article, or claim that may be made by its manufacturer, is not guaranteed or endorsed by the publisher.
